# A new approach to overcoming antibiotic-resistant bacteria: Traditional Chinese medicine therapy based on the gut microbiota

**DOI:** 10.3389/fcimb.2023.1119037

**Published:** 2023-04-06

**Authors:** Peng Xue, Rui Sang, Nan Li, Siyuan Du, Xiuwen Kong, Mingliang Tai, Zhihao Jiang, Ying Chen

**Affiliations:** ^1^ Medical School of Nantong University, Nantong, Jiangsu, China; ^2^ Center for Basic Medical Research, Medical School of Nantong University, Nantong, Jiangsu, China; ^3^ Department of Histology and Embryology, Medical College, Nantong University, Nantong, Jiangsu, China

**Keywords:** traditional Chinese medicine, drug-resistant bacteria, gut microbiota, multi-drug resistance, reversion mechanism

## Abstract

With the irrational use of antibiotics and the increasing abuse of oral antibiotics, the drug resistance of gastrointestinal pathogens has become a prominent problem in clinical practice. Gut microbiota plays an important role in maintaining human health, and the change of microbiota also affects the activity of pathogenic bacteria. Interfering with antibiotic resistant bacteria by affecting gut microbiota has also become an important regulatory signal. In clinical application, due to the unique advantages of traditional Chinese medicine in sterilization and drug resistance, it is possible for traditional Chinese medicine to improve the gut microbial microenvironment. This review discusses the strategies of traditional Chinese medicine for the treatment of drug-resistant bacterial infections by changing the gut microenvironment, unlocking the interaction between microbiota and drug resistance of pathogenic bacteria.

## 1 Introduction

The widespread and irrational use of antibiotics has led to a gradual increase in bacterial resistance to antibiotics and a significant increase in the infection rate of multidrug-resistant bacteria. These strains that are resistant to commonly used antibiotics in clinical practice, called drug-resistant bacteria, are important pathogens for infections in hospitalized patients and have become a serious public health problem. Drug-resistant bacterial infection refers to infectious diseases caused by bacteria that have a certain tolerance to antimicrobial drugs and once drug-resistant bacteria infect occurs, it will not only significantly prolong the patient’s hospital stay, but also have a huge impact on the case fatality rate and overall medical expenses. Recently, the variety of drug-resistant strains has gradually increased, and even “superbugs” have emerged, which has put the already complex resistance mechanism and limited treatment methods in a difficult situation. Therefore, exploring new strategies for the treatment of drug-resistant bacteria has become a very urgent task.

Research into antibiotic resistance is now gradually attracting widespread attention. Chinese traditional medicine (TCM) plays a unique role in regulating the activity of drug-resistant bacteria (Su et al., 2020; Li et al., 2022), including altering the permeability of the biofilm of drug-resistant bacteria, the efflux pump, and the occurrence of resistance mechanisms such as enzyme activity. In addition, most TCMs are taken orally into the digestive tract to exert their regulatory effects on the body, while some studies have found that changes in the gut microbiota and gut microenvironment affect pathogenic bacteria in the gut (Baumler and Sperandio, 2016). Therefore, TCM can also influence the activity of drug-resistant bacteria by regulating the gut microenvironment, indirectly or directly affecting the development of antibiotic resistance. TCM has unique cognition and diagnosis and treatment methods for diseases, in the prevalence of COVID-19, the theoretical system of TCM classifies it into the category of “pestilence”, and its often accompanied symptoms such as low fever, dry cough, and fatigue are related to the “cold and damp” symptoms in the system of TCM (Wang et al., 2021c). TCM provides another unique way of thinking for the prevention and treatment of diseases with its unique cognitive theory. According to the clinical symptoms and characteristics of bacterial infection and drug resistance, infectious diseases are often closely related to a variety of diseases in the TCM system. For example, the production of bacteria and drug resistance often has respiratory symptoms, gastrointestinal symptoms, and so on. In the TCM system, the frequently occurring fever symptoms are classified as “ febrile”, the emerging respiratory symptoms are classified as “cough” and “ chuan syndrome “(asthma), the emerging urinary system symptoms are classified as “stranguria”, and the emerging digestive tract symptoms are classified as “diarrhea”. The understanding of drug resistant bacteria infection in TCM theory is related to the “six evil factors”, “internal injury fever”, “deficiency of vital qi”, and “ hidden pathogen” in TCM theory. This review will summarize the infection mechanism of drug-resistant bacteria, the characteristic advantages of TCM in regulating the gut microenvironment for the treatment of drug-resistant bacterial infections, and the TCM treatment of commonly used drug-resistant bacteria, to find effective TCM strategies for the treatment of drug-resistant bacterial infections. At the same time, it provides theoretical support for dialectical treatment and individualized treatment of TCM.

## 2 Mechanisms of antibiotic resistance

**Figure 1:**
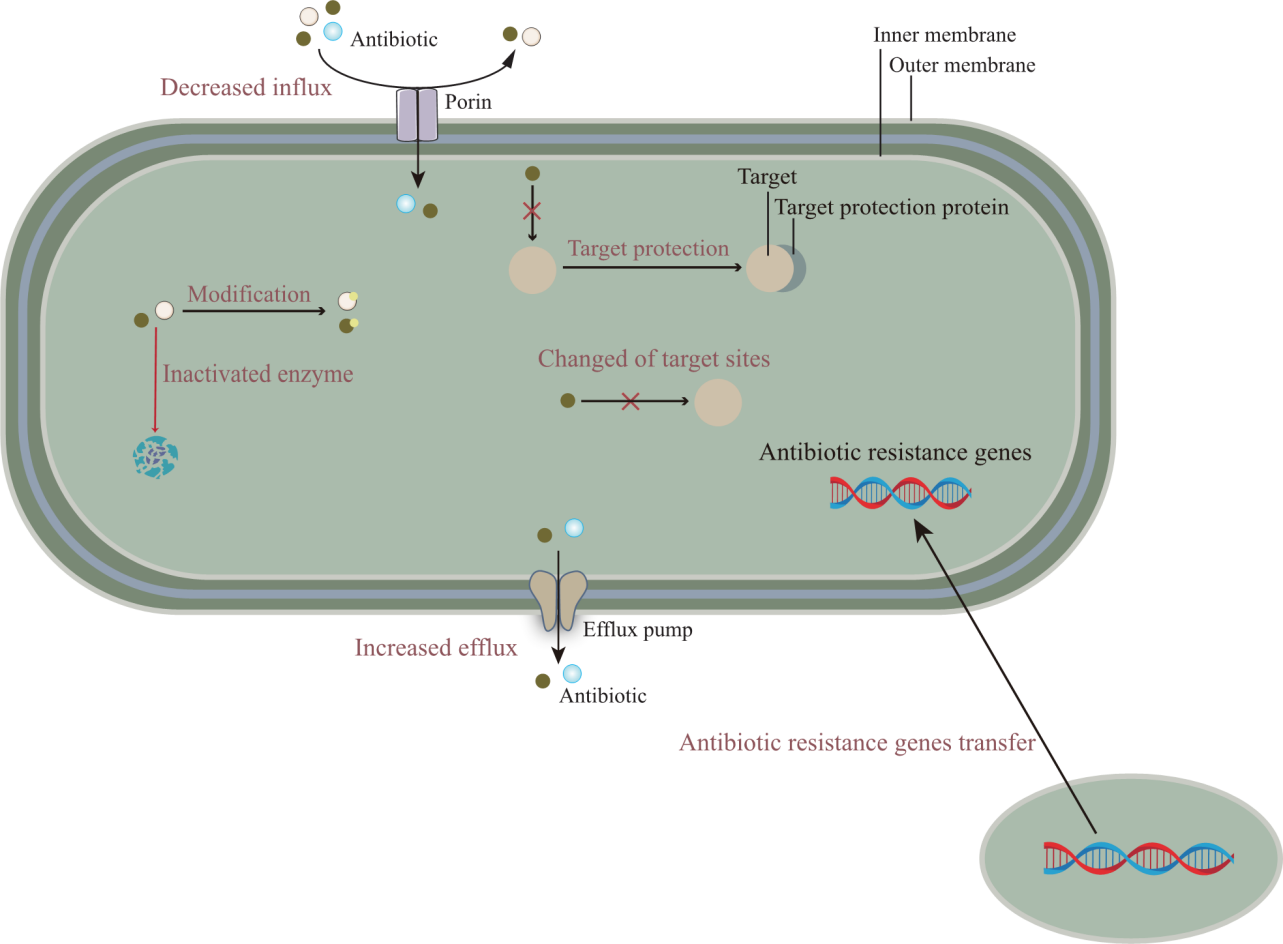
The molecular mechanisms of antibiotic resistance are mainly composed of the following: (i) Reducing the entry of antibiotics, that is, down-regulation of porin expression; Or promote the efflux pump to discharge antibiotics. (ii) The resistance gene is transferred from other pathogens to the target bacteria to enhance the antibiotic resistance of the target bacteria. (iii) The mutation of gene target site reduces the affinity between antibiotics and target sites or cannot combine with them. In addition, related proteins can also protect target sites and prevent the binding of antibiotics. (iv) Antibiotics are modified or degraded by related enzymes.

The emergence of multidrug-resistant bacteria increases morbidity and mortality in infected patients, which has a huge negative impact on the prognosis of many groups and imposes an economic burden on society (Albrich et al., 2004). Clinically, antibiotic therapy is the first choice for the treatment of bacterial infectious diseases (Michael et al., 2014; Naylor et al., 2018). However, due to the irrational use of antibiotics lead to the gradual development of bacterial resistance, so the current commonly used antibiotic treatment is not effective. Combined with its own structural characteristics, bacteria have a variety of drug resistance mechanisms. The development of bacterial antibiotic resistance is associated with a variety of pathways (Munita and Arias, 2016), including, in general, a genetic basis and a variety of biochemical pathways, such as mutations in resistance genes, modification of antibiotic molecules, and changes in bacterial membrane structure, **Figure 1** describes the relevant mechanisms.

### 2.1 Gene mutations

Mutation and horizontal gene transfer (HGT) may be specific reasons for the emergence of bacterial resistance (Martinez and Baquero, 2000; Mouton et al., 2012). Mutations that cause antibiotic resistance typically occur in three types of genes: genes encoding antibiotic targets, genes encoding their transporters, and genes encoding transporters that inhibit them. Mutations in these genes contribute to the acquisition of antibiotic resistance. The efflux system is widespread in gram-positive bacteria such as Staphylococcus aureus; Gram-negative bacteria, such as Escherichia coli, Pseudomonas aeruginosa, and Acinetobacter baumannii, whose role is to excrete the drug from the bacteria. Often synergistic with altered permeability of the bacterial outer membrane, leading to the development of bacterial multidrug resistance (Li and Nikaido, 2009; Coyne et al., 2011).

### 2.2 Changes in the structure of the biofilm of bacteria

Bacterial biofilm is a natural form of bacterial growth that attaches to the surface of tissues and is embedded in the flora in the extracellular matrix, which is a way for drug-resistant bacteria to adapt to their living environment (Masak et al., 2014). Due to the presence of biofilms, pathogens can evade immune attack from the host, making it difficult for pathogens to be cleared, thus forming chronic foci of infection (Li and Lee, 2017). Studies have shown that biofilms prevent antimicrobials from entering biofilms through their dense structure and intermolecular interactions, thereby increasing the resistance of pathogenic bacteria (Djeribi et al., 2013). In addition to its own structural characteristics, after bacteria form a biofilm, the submembrane bacteria metabolism is low, which can reduce the permeability of antibacterial drugs and promote their hydrolysis, resulting in resistance to antibacterial drugs (Davies, 2003).

### 2.3 Passivation enzyme or inactivated enzyme production

Passivation enzymes or inactivated enzyme mechanisms can lead to structural changes or inactivation of antimicrobial drugs, which in turn disrupt the action of various antimicrobial drugs, and antibiotic resistance is not only the result of the action of a few specific elements, but the result of the overall physiology of bacteria. At present, the inactivated or passivation enzymes produced by bacteria are mainly β-lactamase, aminoglycoside antibacterial drugs passivation enzyme, chloramphenicol acetyltransferase, etc., of which β-lactamase is the most widely studied (Hartman and Tomasz, 1984), and most antibiotics affected by these enzyme modifications work by inhibiting protein synthesis at the ribosome level (Wilson, 2014).

### 2.4 Cyclic di-GMP signaling pathway

Cyclic di-GMP (c-di-GMP) is a second messenger molecule commonly found in bacteria and high levels of c-di-GMP reduce flagellar activity and increase the expression of adhesin and extracellular polysaccharides (Hengge, 2009). At the same time, the level of c-di-GMP also affects the growth and metabolism rate of bacteria, membrane permeability, and other factors related to the formation of biological coatings (Chua et al., 2013).

### 2.5 Changes in the target of action of antimicrobial drugs

Bacteria can also lead to antibiotic resistance by changing the physiological importance of the target site. This mechanism is mainly regulated by bacterial DNA gyrase and topoisomerase, encoded by gyrA and gyrB genes, grl A and grl B genes, respectively (Cheng et al., 2007). Once the gene of the target enzyme is mutated, it will lead to a decrease in the effect of antibacterial drugs, such as β-lactam antibacterial drug target change and vancomycin action target change. This resistance mechanism is less specific for antimicrobial drugs, making it more difficult to select effective drugs in clinical practice.

## 3 Common classification of resistant bacteria in gut

Due to the widespread use of antibiotics and immune preparations, the drug resistance of clinically isolated bacteria is more common than usual and the drug resistance rate is still on the rise, among which methicillin-resistant Staphylococcus aureus (MRSA) and extended-spectrum β-lactamase (ES-BLs) Enterobacteriaceae bacteria have caused serious difficulties in clinical treatment. Therefore, it is of great significance to be familiar with the resistance mechanism of various common drug-resistant bacteria and correctly apply antibiotics to prevent and control bacterial drug resistance and improve clinical efficacy. In this part and **Table 1**, the common drug-resistant bacteria in the intestinal tract will be specifically described according to the two categories of gram-positive bacteria and gram-negative bacteria.

**Table 1:** Common drug-resistant bacteria and its mechanism.

Type	Organism	Resistance mechanism	Drugs treatment	References
G+	*Methicillin-resistant staphylococcus*	Production of β-lactamase Penicillin-binding protein 2a	Vancomycin Ampicillin Zocillin, etc.	(Henson et al., 2017) (Goel et al., 2021) (Tasneem et al., 2022)
G+	*Enterococcus*	Production of PBP5 Alteration of membrane permeability Aminoglycoside blunting enzymes	Vancomycin Piperacillin sodium	(Montealegre et al., 2017) (Tran et al., 2013) (Miller et al., 2020)
G-	*Helicobacter pylori*	Genetic mutations	Amoxicillin	(Tran et al., 2022)
G-	*Escherichia coli*	Efflux Pumps	Hydrophobic fluoroquinolones	(Huguet et al., 2013)
G-	*Acinetobacter baumannii*	Structural changes in target proteins Absence of outer membrane proteins and changes in membrane permeability	Cefadroxil Ampicillin sodium sulbactam sodium	(Lee et al., 2017) (Skariyachan et al., 2019)
G-	*Serovar Typhimurium*	Antibiotic resistant plasmid transfer	–	(Bakkeren et al., 2019)

### 3.1 Gram-positive bacteria

#### 3.1.1 Methicillin-resistant staphylococci

Methicillin-resistant staphylococci is divided into MRSA, methicillin-resistant Staphylococcus epidermis (MRSE), and methicillin-resistant Staphylococcus haemolyticus (MRSH). A large-scale bacterial resistance test in China showed that the average methicillin-resistant strains of staphylococcus aureus and coagulase-negative staphylococci isolated in 14 hospitals in 10 provinces and cities were 51.7% and 71.6% (Zhu et al., 2011). MRSA is a major problem of bacterial resistance because MRSA is cross-resistant to β-lactam drugs and often synergistic to aminoglycosides, macrolides, and quinolones (Vestergaard et al., 2019).

#### 3.1.2 Enterococcus

Enterococcus is widely found in soil, water, and other natural sources, is one of the components of the normal gut microbiota, and is the most common gram-positive coccus that causes nosocomial infections in addition to staphylococcus. The bacterium is a facultative anaerobic bacterium with the characteristics of salt tolerance, high temperature resistance, and wide temperature range of growth environment, which can lead to urinary tract infectious diseases, infective endocarditis, complex skin infections, bacteremia, etc (O'Driscoll and Crank, 2015). Therefore, screening enterococcal strains with high resistance to aminoglycosides, namely gentamicin MIC≥500μg/ml, streptomycin MIC≥ 1000μg/ml, is of great value for guiding clinical medication.

#### 3.1.3 Streptococcus pneumoniae

Streptococcus pneumoniae is the most common pathogen of respiratory infections, and drug resistance rates have increased rapidly in recent years. Insensitivity to penicillin and high macrolides are major resistance problems in Streptococcus pneumoniae. The main resistance mechanisms are as follows: the resistance mechanism of penicillin-resistant Streptococcus pneumoniae (PRSP) is mainly due to the bacteria’s penicillin-binding protein (PBP) and is not related to plasmid-mediated β-lactamase (Goh et al., 2002; Pagliero et al., 2004). The study showed that mice infected with Streptococcus pneumoniae after disruption of normal gut microbiota had more bacteria in their blood and increased lung inflammation. Timely restoration of lung bacterial clearance and improvement of inflammatory symptoms after faecal microbiota transplantation (Schuijt et al., 2016). We therefore suggest that disruptions in the structure of the gut microbiota may lead to changes in the drug resistance of pathogenic bacteria.

### 3.2 Gram-negative bacteria

#### 3.2.1 Helicobacter pylori

*Helicobacter pylori* is a microaerobic bacterium, discovered by Barry Marshall in Australia in 1982, and a large number of studies have confirmed that *Helicobacter pylori* is the main cause of chronic gastritis, gastric ulcer and duodenal ulcer, and is closely related to the occurrence of gastric cancer and gastric mucosa-associated lymphoid tissue lymphoma, and more than 6% of cancers worldwide and about 90% of non-cardia gastric cancer cases are attributed to *Helicobacter pylori* infection (O'Connor et al., 2017). Therefore, in 1994, the World Health Organization’s International Agency for Research on Cancer classified the bacterium as a class of carcinogens. Most of the treatment methods are antibiotic therapy combined with proton pump inhibitors, but due to the gradual increase of drug resistance, the treatment effect is not obvious. *Helicobacter pylori* is primarily acquired, and genetic mutations are the main way that causes *Helicobacter pylori* to nitroimidazole (metronidazole) resistance.

#### 3.2.2 Escherichia coli and Klebsiella pneumoniae

##### 3.2.2.1 Production of β-lactamase

β-lactamase is one of the main causes of resistance of gram-negative bacteria to β-lactam antibiotics. There are four clinically important β-lactamase: AmpC enzymes, ESBLs, β-lactamase that hydrolyzes carbapenems, and β-lactamase that is resistant to enzyme inhibitors. The most important are ESBLs and AmpC enzymes. AmpC enzymes represent a family of cephalosporins, produced by gram-negative bacteria that are not inhibited by clavulanic acid. About 40% of Enterobacteriaceae genus can produce high AmpC enzymes (Martin et al., 2018). Escherichia coli and Klebsiella pneumoniae produce ESBLs, the resistance of which is mainly mediated by plasmids. In addition, ESBLs-carrying genes often carry resistance genes such as aminoglycosides, quinolones, and chloramphenicol, resulting in multi-drug resistance. Strains that produce AmpC-inducing enzymes have been found to produce ESBLs at the same time. For example, Escherichia coli and Klebsiella pneumoniae can produce ESBLs and AmpC at the same time, the former with an incidence of 2% and the latter with 17.1%. Bacteria that produce both ESBLs and plasmid-type AmpCases are called ultra-extended-β-lactamases, which are more resistant, more transmissive, and more difficult to control clinically appropriate infections.

##### 3.2.2.2 Change in target location

Almost all gram-negative bacteria contain PBPs, which are produced by bacterial cell membranes. Binding of E. coli to β-lactam antibiotics can lead to bacterial morphological changes, β-lactam antibiotics binding to PBP1 will cause bacterial autolysis, binding to PBP2 will cause spherocytes, and binding to PBP3 will cause bacterial filamentous changes.

##### 3.2.2.3 Alteration of membrane permeability

Susceptible bacteria with a hyperosmolic outer membrane can become resistant to antibiotics by reducing the permeability of the outer membrane. For example, OmpF mutant strains of Escherichia coli are resistant to fluoroquinolone antibiotics (Poirel et al., 2018). The reduced permeability of the membrane can strengthen the enzyme inactivation system, and the outer membrane barrier has a synergistic effect with β-lactamase, which is also the cause of multidrug resistance.

##### 3.2.2.4 Formation of biological envelope

The formation of bacterial film enhances the adhesion ability of bacteria to tissues, and reduces the penetration of antibiotics and enhances drug resistance; It can adsorb the antibacterial drug passivation enzyme and promote the hydrolysis of antibacterial drugs; Make the body immune escape to bacteria; Reduced sensitivity to antibacterial drugs.

#### 3.1.3 Pseudomonas aeruginosa

Pseudomonas aeruginosa is the most common bacterium among non-fermented gram-negative bacilli, ranking 2nd among negative bacilli. Clinically, multidrug resistance and pan-drug resistance, especially resistance obtained under the pressure of antibiotic selection, are more clinically harmful.

#### 3.1.4 Maltophilus stenophilus

Maltophilus stenophilus, formerly known as “*Pseudomonas maltophila*”, was classified into the genus Xanthomonas in 1983, called “*Xanthomonas maltophila*”, because the metalloenzyme produced by Maltophilus oligoxophilus can hydrolyze carbapenem antibacterial drugs, which are naturally resistant to imipenem and meropenem, and its outer membrane permeability is low, and a variety of β-lactamases are produced; Therefore, multi-drug resistance is shown against antimicrobial drugs.

## 4 Clinical treatment of drug-resistant bacteria

In this section, we will summarize common treatment modalities for clinically resistant infections in the emerging stage. **Table 2** summarizes the clinical treatment methods and new methods of common drug-resistant bacteria.

**Table 2:** Treatment methods and new methods of drug-resistant bacteria.

Organism	Treatment	New methods	References
*Methicillin-resistant staphylococcus*	Vancomycin	Fifth generation cephalosporins, New oxazolidinones, New generation fluoroquinolones, Lipoglycopeptides, Novel tetracycline-derivatives, Lefamulin	(Vena et al., 2022)
*Carbapenem-resistant Enterobacteriaceae*	Meropenem, Amikacin, Polymyxin B	New antibacterial agents, Nanoparticles, Phage therapy, Vaccine	(Kulengowski et al., 2018) (Tilahun et al., 2021)
*Vancomycin-resistant Enterococcus*	Linezolid, Daptomycin	Stachyose, Methylsulfonylmethane	(Twilla et al., 2012) (Zhu et al., 2021) (Poole et al., 2021)
*Multidrug-resistant Pseudomonas aeruginosa*	Imipenem-Relebactam, Cefepime-Zidebactam, Cefiderocol, Murepavadin	Silver nanoparticles	(Horcajada et al., 2019) (Liao et al., 2019)
*Multidrug-resistant Escherichia coli*	Mecillinam	Natural compounds, Nanoparticles	(Zykov et al., 2020) (Masota et al., 2022) (Khare et al., 2021)

### 4.1 Combination of antibiotics

Bacteriophage therapy is already considered as a potential treatment for drug-resistant pathogens, and its combination with antibiotics can be used to enhance the eradication of drug-resistant pathogens and alleviate widespread antibiotic resistance worldwide. This combination therapy has been shown to have the advantages of enhanced bacterial inhibition, more effective penetration into biofilms, and reduced chance of phage resistance (Tagliaferri et al., 2019; Li et al., 2021).

### 4.2 Research and development of new antibiotics

Because the research of new antibiotics is time-consuming and costly, it is not easy for new antibiotics to appear in a short period of time, so finding new drugs that can replace antibiotics has become a top priority. Relevant studies have shown that cephalosporins, oxazolidinone, omada ring, glycopeptides: darbavaromycin, Teixobactin new antibiotics, arachidonic acid protein, antimicrobial peptides, etc., these substances have been found to have antibiotic-like functions (Karaman et al., 2020).

### 4.3 New methods of treating drug-resistant bacteria

#### 4.3.1 Phages

Compared with antibiotics, bacteriophages have high specificity, small dose, low production cost, high safety and high antibiotic envelope activity (Abdelkader et al., 2019; D'Accolti et al., 2021). In order to cope with the increasingly serious threat of drug-resistant bacteria, phage therapy has been proposed as an alternative to antibiotic therapy. Also known as bacterial viruses, bacteriophages are widely distributed in nature and can infect and kill bacteria (Comeau et al., 2008). The specific mechanism is that bacteriophages can eradicate bacterial biofilms, which penetrate deep into the biofilm by using water channels within the biofilm, or by destroying the extracellular biofilm matrix by expressing depolymerase (Parasion et al., 2014; Kortright et al., 2019).

#### 4.3.2 Probiotics

As a new treatment model, probiotics improve the function of antibiotics by producing antimicrobial compounds such as bacteriocins, hydrogen peroxide, nitric oxide and short-chain fatty acids, thereby reducing the number of pathogenic bacteria and destroying biofilms, and also by reducing the co-aggregation of pathogenic bacteria, adhesion to cells and the production of organic acids antagonizing pathogenic bacteria. It is definite is that probiotics can be used as a partial substitute or adjunct to antibiotic therapy to help treat multidrug-resistant infections (Ouwehand et al., 2016; Newman and Arshad, 2020).

#### 4.3.3 Nanotherapy

The presence of bacterial biofilms is an important factor leading to the development of drug resistance in bacteria. Due to their small particles and large specific surface area, bacterial metal nanoparticles greatly increase the contact surface with bacteria, so they have strong antibacterial activity (Stefan et al., 2011; Palanisamy et al., 2014). At the same time, its antibacterial effect has multi-target characteristics, which can change cell permeability, and can also interfere with the function of sulfur-containing proteins and phosphorus-containing compounds (such as DNA), making it difficult for bacteria to develop drug resistance (Raghunath and Perumal, 2017). However, because metal nanoparticles can have certain toxic side effects on the human body, they are less used in clinical practice (Lewinski et al., 2008), and there are currently metal nanoparticles used in combination with antibiotics to reduce the amount of metal nanoparticles and make it possible to reduce toxicity.

## 5 Research on the effect of traditional Chinese medicine on common drug-resistant bacteria in gut

**Figure 2:**
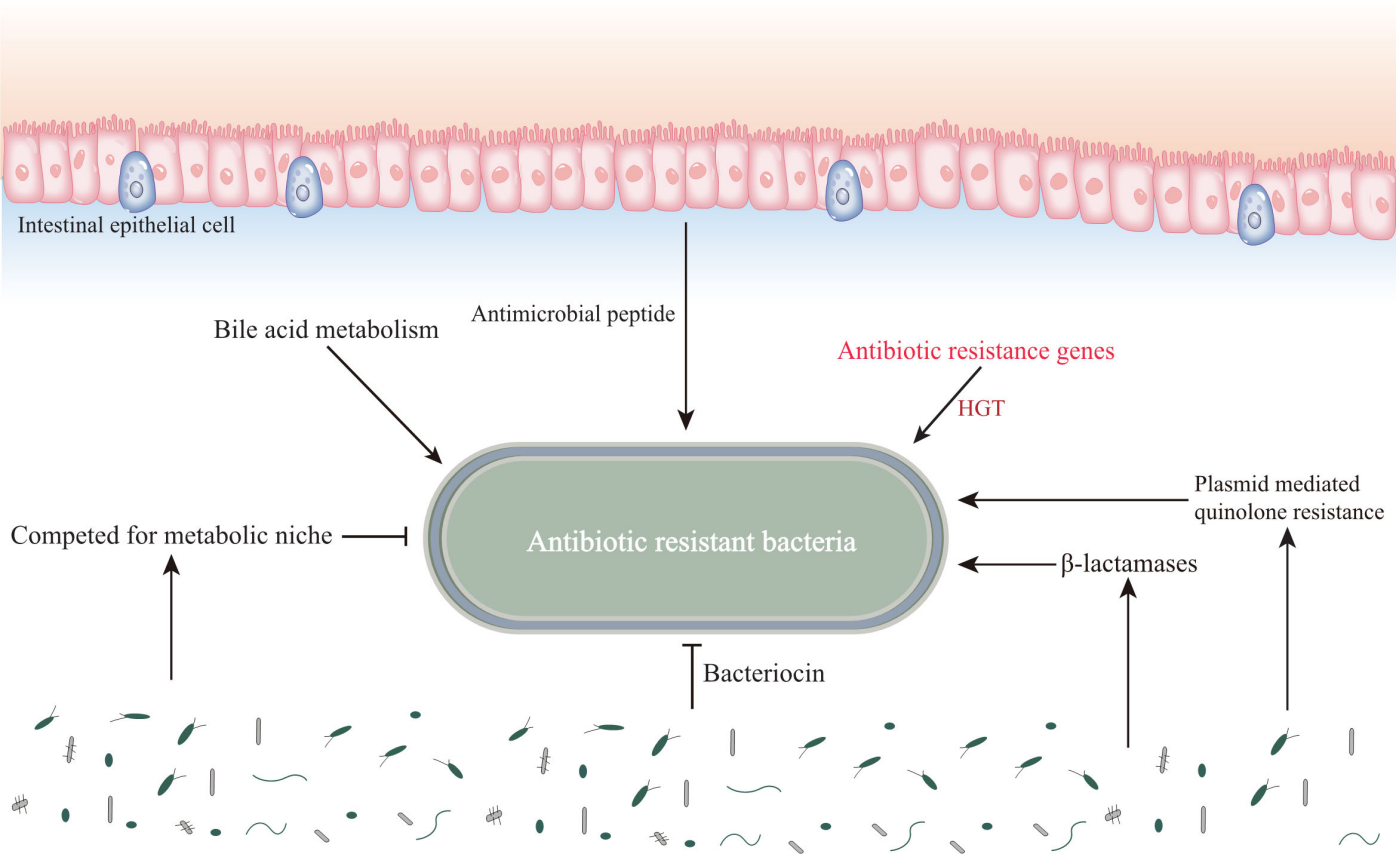
Influence of gut microenvironment on common drug-resistant bacteria. The gut microenvironment stores resistant substances, which affects the drug resistance of pathogenic microorganisms. In addition, the diversity of intestinal microbiota affects the colonization and proliferation of drug resistant bacteria.

We have previously understood that resistance in resistant bacteria is largely due to the extensive use of antibiotics. Until now, the untapped chemical diversity in traditional herbal medicine has been overlooked, and TCM has a long history and rich experience in treating infectious diseases. Many studies have also shown that traditional Chinese medicine has significant antibacterial or bactericidal effects, and the main mechanisms include inhibition of biofilm formation of drug-resistant bacteria, efflux pump system, enzyme activity, and changes in bacterial permeability (Su et al., 2020) (**Figure 2**). Below we will elaborate on the unique advantages of traditional Chinese medicine in the treatment of drug-resistant bacteria (**Table 3**).

**Table 3:** Effect and mechanism of TCM and its effective components on antibiotic resistant bacteria.

TCM Herbs	Mechanism of action	Drug resistant strains	References
Luteolin	Membrane disrupted	MRSA	(Zhang et al., 2018)
Geraniol	Reduction of Membrane; Increased host immune response sensitivity	GCC; MRSA	(Kannappan et al., 2019)
Berberine	Inhibition of ERG11 and efflux pump expression	*Candida tropicalis*	(Shi et al., 2017)
Epigallocatechin-3-gallate	Reduction of Membrane	MRSA	(Knidel et al., 2021) (Zeferino et al., 2022)
Hyperoside	Inhibited Salmonella T3SS need protein InvG	*Salmonella enterica*	(Zhang et al., 2022)
Sophoraflavanone G	Inhibited NorA efflux pump	MRSA	(Sun et al., 2020)
Gallic acid	Inhibited membrane permeability	*Escherichia coli*	(Tian et al., 2022)
Patchouli Alcohol	Downregulated gene expression of efflux pump	*Helicobacter pylori*	(Zhong et al., 2021)
Isovalerylshikonin	Reduced msrA expression	Drug-resistant *Staphylococcus aureus*	(He et al., 2019)
Eugenol	Inhibited membrane; Destroyed intercellular connection	MRSA	(Yadav et al., 2015)
Cryptotanshinone	Obstructed bacterial energy metabolism	MRSA	(Zhong et al., 2021)
Bisdemethoxycurcumin	Inhibited membrane; Inhibited the expression of Virus Related Exoproteins	MRSA	(Wang et al., 2021a)
Baicalein	SOS reaction inhibitor	*Staphylococcus aureus*	(Peng et al., 2011)
Sodium new houttuyfonate; berberine chloride	Cell membrane rupture	*Staphylococcus aureus*	(Li et al., 2020)
Berberine, curcumin	Inhibited membrane	MRSA	(Bhatia et al., 2021)
Carvacrol, thymol	Inhibited NorA efflux pump	*Staphylococcus aureus*	(Dos et al., 2021)
emodin, baicalin, schizandrin and berberine	Downregulated the expression of hefA	*Helicobacter pylori*	(Huang et al., 2015a)
Water extract of Aloe vera	Inhibited membrane permeability	*Escherichia coli*	(Wu et al., 2022)
Magnolia officinalis, Verbena officinalis, Momordica charantia, and Daphne genkwa	PBP inhibitor	MRSA	(Kuok et al., 2017)
Ginkgo biloba L.	Downregulated the virulence gene hld; Membrane disrupted	MRSA	(Wang et al., 2021b)
Portulaca oleracea L.	Inhibited efflux pump	MRSA	(Chan et al., 2015)
Schisandra chinensis (Turcz.) Baill	Metalo-β-lactamase inhibitor	*Bacillus cereus; Aeromonas hydrophila*	(Duan et al., 2021)
Psoralea corylifolia	Membrane damage; Energy consumption	*Listeria monocytogenes;* MRSA	(Li et al., 2019)
Morus alba	Destroyed proton power and membrane permeability	MRSA	(Wu et al., 2019)
Jinghua Weikang capsule	Inhibited efflux pump, inhibited membrane, reduced adhesin	*Helicobacter pylori*	(Jia et al., 2022)
SanHuang Decoction	Inhibited membrane, reduced adhesin	*Staphylococcal strains*	(Zhang et al., 2021)
Qixingjian Decoction	Inhibited the expression of virulence factors, destroyed cell wall	MRSA	(Lv et al., 2022)

### 5.1 The role of gut microbiota in antibiotic resistance

Various pathogenic bacteria accumulate in the human gut, which in turn can interact with the gut microenvironment to influence their resistance under certain circumstances, such as HGT of the gut microbiota, colonization resistance and destruction, and the influence of stored substances in the gut. *Vibrio cholerae*, and *Salmonella typhimurium* can acquire drug-resistant genetic material directly from the gut and insert it into their genomes, a process that may be closely related to the involvement of Escherichia coli (Bakkeren et al., 2019; Mcinnes et al., 2020). In addition, drug-resistant substances produced by gut bacteria can be transferred to pathogenic bacteria *via* cellular microvesicles, for example, the *Bacteroides* spp. produces membrane vesicles carrying surface-associated β-lactamases that can protect commensal bacteria and intestinal pathogens from β-lactam antibiotics (Stentz et al., 2015). The human gut also contains a large community of phages carrying antibiotic resistance genes, for example, phages in the feces of ciprofloxacin-resistant mice carry genes encoding quinolone efflux pumps, while phages in the feces of ampicillin-resistant mice carry genes encoding sensors for cell wall synthesis inhibitors and response regulators, suggesting that phages in the intestine transfer antibiotic resistance genes into pathogens to enhance their resistance (Shuwen and Kefeng, 2022). The normal gut microbiota is resistant to colonization and proliferation of pathogens, including drug-resistant bacteria. However, irrational antibiotic use disrupts this colonization resistance, and studies have shown that single doses of clindamycin reduce gut microbiota diversity and accelerate the development of diarrhea and colitis (Buffie et al., 2012). In addition, the gut itself acts as a reservoir for β-lactamases and plasmid-mediated quinolone resistance in β-lactam and quinolone drug resistance (Anthony et al., 2021).

### 5.2 Effects of natural compounds and monomers of traditional Chinese medicine on different drug resistant bacteria

#### 5.2.1 Helicobacter pylori

*Helicobacter pylori* is an important pathogenic factor of chronic gastritis and peptic ulcer, and Asia is a region with a high incidence of *Helicobacter pylori*, so timely control of *Helicobacter pylori* has become the primary goal. Clinically, quasi-triple therapy is often used, that is, the use of proton pump inhibitors or ranitidine bismuth citrate, combined with clarithromycin, amoxicillin or metronidazole to inhibit gastric acid secretion, antibacterial, and protect the gastric mucosa. However, in recent years, due to the emergence of drug resistance, the eradication rate has continued to decline, so new treatments are being implemented (Dang et al., 2020). TCM treatment emphasizes the overall concept and care for the spleen and stomach, pays attention to promoting the regulation of human systemic immunity, protects local defense factors of tsahe gastrointestinal mucosa, and improves the quality of mucosal repair, so it has the characteristics of efficacy stability and small drug resistance (Liu et al., 2018). Many single herbs have anti-*Helicobacter pylori* effects, and have a strong inhibitory effect on *Helicobacter pylori* in the body by highly sensitive drugs such as Chuanglian, Skullcap, Panax notoginseng, etc. Bacterial biofilm is a viscous substance secreted by many bacteria gathered and bonded together, mainly polysaccharides, nucleic acids, lipids and proteins, etc., intertwined and adhered to abiotic or biological surfaces to form a “film”. Biofilms can protect bacteria against external dangers, such as enhancing resistance to antimicrobial drugs, which can make treatment difficult and make infections recur (Chen et al., 2020). H. pylori, which forms biofilms, is highly resistant and evades attacks from the immune system, and its infection is prone to chronicity and difficult to control. Studies have shown that by observing the effect of TCM extracts on the formation of drug-resistant *Helicobacter pylori* biofilm, it is found that emodin, berberine, matrine and baicalin have inhibitory effects on drug-resistant *Helicobacter pylori*, and the concentration <50% minimum inhibitory concentration can significantly inhibit the formation of strain biofilm. The possible mechanism is that some strains are inhibited, and the capsule of the surviving strain is destroyed, resulting in a decrease in its ability to secrete polysaccharides and affecting the adhesion of the strain (Huang et al., 2015a). Skullcap is the dried root of skullcap, first recorded in the “Shennong Materia Medica”. The main active ingredients of skullcap are flavonoids, of which baicalin and baicalin can relieve gastrointestinal dysfunction caused by western medicine (Mehendale et al., 2007). An *in vitro* study found that baicalin and baicalensis ethanol extract had the bactericidal effect on *Helicobacter pylori* and the antibacterial activity of baicalin was greater than that of baicalensis extract, revealing that the combination of baicalensis has great research prospects in the future (Wu et al., 2008).

#### 5.2.2 Methicillin-resistant Staphylococcus aureus

Methicillin-resistant Staphylococcus aureus is the most common human pathogen of antibiotics and is commonly treated with vancomycin, but has poor efficacy due to the development of resistance. The use of traditional Chinese medicine has brought a new perspective to the treatment of drug-resistant bacteria. MRSA or Staphylococcus aureus infections against penicillinase can be treated with a combination of baicalin and penicillin. At the same time, it has been shown that linezolid and baicalein can inhibit the formation of biofilms in the body, thereby exerting anti-MRSA effects (Qian et al., 2015; Liu et al., 2020b). Synergistic effect of luteolin and quercetin in combination with imipenem and ceftriaxone on MRSA. In addition, the combination of luteolin with ampicillin, zacillin and gentamicin can synergistically enhance the antibacterial effect of aminoglycosides and β-lactam antibiotics on MRSA (Joung et al., 2016). Houttufen sodium and berberine sodium chloride eradicate growth and persistent resistant Staphylococcus aureus (Li et al., 2020). Flavonoids, which are widely distributed plant compounds such as baicalin, have been found to exert antimicrobial effects when used in combination with tetracyclines and β-lactam antibiotics that significantly reduce the MIC of MRSA (Fujita et al., 2005). Some studies have shown that the combination of magnolia, horsetail, bitter melon and coriander extracts with antibiotics has found that the minimum inhibitory concentration of methicillin-sensitive Staphylococcus aureus decreased and the fractional inhibitory concentration index increased (Kuok et al., 2017). Geraniol is a cyclic monoterpene alcohol found naturally in essential oils of lemon, lemongrass, lavender. In Kannappan’s study, Caenorhabditis elegans was used as a staphylococcus aureus vector. The study confirmed that the combination of geraniol and cefotaxime was more effective in inhibiting the formation of BF by pathogens compared with monotherapy or untreated controls (Kannappan et al., 2019).

#### 5.2.3 Staphylococcus epidermidis

Staphylococcus epidermidis is a microbiota that usually lives on the surface of normal human skin, and is generally not pathogenic, but can be accompanied by molecular materials entering the human body under special circumstances such as interventional treatment, and can also cause sepsis after invading blood circulation, causing serious consequences (Skiba-Kurek et al., 2021). KANNAPPAN also did the same *in vivo* research on Staphylococcus epidermidis, and under the treatment of different combinations of geraniol and cefotaxime, it can effectively inhibit the formation of BF by pathogens (Kannappan et al., 2019).

#### 5.2.4 Candida albicans

Candida albicans is an opportunistic pathogen and the most common pathogen of invasive candidiasis, and its BF is an important reason for the development of drug resistance of Candida albicans (Lee et al., 2021). YHN is a derivative of andrographolide, the main active component of natural plant andrographis, which has the effect of removing heat and detoxification, anti-inflammatory and analgesic. In a study modeled on the subcutaneous duct of Candida albicans rats, it was shown that potassium sodium salt can play an effective inhibitory role in the formation of Candida albicans BF and is concentration-dependent. At the same time, YHN can significantly reduce the ability of Candida albicans to adhere to the duct, which is likely to be one of the reasons for reducing the formation of Candida albicans BF in rats. (Effect of andrographolide derivative YHN on Candida albicans biofilm in rats) At the same time, it has been found that berberine can promote the biosynthesis of ergosterol, thus making it resistant to Candida albicans. Fluconazole in combination can eliminate berberine resistance and synergize with berberine against drug-resistant Candida albicans. Moreover, berberine chloride also has a certain antibacterial effect on oral streptococcus (Dziedzic et al., 2015; Shi et al., 2017). In addition, eugenol in combination with less toxic amphotericin B can effectively enhance the antifungal effect and reduce the ability to reduce toxicity (Khan et al., 2019).

#### 5.2.5 Pseudomonas aeruginosa

*Pseudomonas aeruginosa* is an aerobic gram-negative bacterium that is opportunistic pathogenic and one of the common pathogenic bacteria susceptible to immunocompromised people such as hospitalized patients (Aldawsari et al., 2021). Allicin is one of the main active ingredients of garlic, which has different degrees of killing and inhibiting effects on a variety of pathogenic microorganisms. In this respect, BJARNSHOLT found that garlic extract increased the sensitivity of *Pseudomonas aeruginosa* to tobramycin, respiratory bursts, and neutrophil phagocytosis in a mouse model of lung infection (Bjarnsholt et al., 2005).

#### 5.2.6 Mycobacterium tuberculosis

It has been shown that TCM compounds containing polyglutamic acid synthase such as yaconamide C, methyl 3-o-feruloylquineate can reduce mycobacterial tuberculosis drug resistance by inhibiting the folC transcription protein (Folylpolyglutamate synthetase) of the drug resistance related gene folC of Mycobacterium tuberculosis (Hung et al., 2014).

#### 5.2.7 Escherichia coli

Many scholars have found that the antibacterial activity of fluoroquinolones against multidrug-resistant Escherichia coli is enhanced. silver nanoparticles synthesized by green method using forsythia fruit aqueous extract as raw material also have certain antibacterial activity against Escherichia coli (Du et al., 2019).

### 5.3 Combined use of antidrug-resistant bacteria in traditional Chinese medicine

Compared with single TCM, the combined use of various TCMs has a more obvious inhibitory effect on bacteria. This treatment method has long been widely used in TCM treatment, and a variety of compound preparations have been derived. In the Han Dynasty’s Zhang Zhongjing’s “On Typhoid Fever”, it is recorded that Banxiaxiexin Decoction, which is a representative remedy for the treatment of gastrointestinal disorders caused by stomach and spleen deficiency, unsmooth lifting, cold and heat obstruction, and vomiting. Banxia, skullcap, dried ginger, licorice, ginseng, huanglian, and jujube together make up Banxiaxiexin Decoction. In a study of 30 patients with Banxiaxiexin decoction combined with triple therapy, the observed *Helicobacter pylori* eradication rate was 90.00%, which was significantly higher than that of the triple therapy group. Banxiaxiexin Decoction may act as an antidrug-resistant bacterium by inhibiting the *Helicobacter pylori* toll-like receptor (TLR)/nuclear factor-κB (NF-κB) signaling pathway (Wu et al., 2019). Ditan Decoction is mainly composed of ginger, southern star, banxia, citrus aurantium, poria, orange, calamus, ginseng, bamboo ru and licorice, which has the effects of dissolving phlegm, reducing fire, ventilating and enhancing physical fitness. Some scholars have observed the clinical efficacy of Ditan Decoction on superdrug-resistant bacteria, and the results show that Ditan Decoction has a good inhibitory effect on Streptococcus pneumoniae, Haemophilus influenzae, Staphylococcus aureus, Escherichia coli, Pseudomonas aeruginosa, Acinetobacter baureus, etc., with high antibacterial activity, which can well inhibit the activity of superdrug-resistant bacteria forming enzymes (Huang et al., 2015b) The ingredients of FuFangJinQianCaoQingReKeLi are mountain sesame, gourd tea, maran grass, centella asiatica, dog liver vegetables, fence net, jade leaf golden flower, ground gall grass, money grass, cloth residue leaf, licorice, gangmei, with wind relief table, clear heat and detoxification. A study reported that the pharmacodynamic effect of FuFangJinQianCaoQingReKeLi on acute drug-resistant bacterial infection, and the results showed that the compound heat-clearing granules could improve the general condition of animals infected with acute drug-resistant bacteria, reduce plasma endotoxins, regulate the content of anti-inflammatory and pro-inflammatory cytokines, increase the neutrophil phagocytosis index, reduce the pathological damage of organ tissues of model animals, and reduce the mortality rate (Yang et al., 2009).

### 5.4 Other treatment

In recent years, more Chinese herbal ingredients have been discovered to have the potential to treat drug-resistant bacteria. For example, the antibacterial activity of cephalogram extract and its structural components (Liao et al., 2011). The isolation of FSP from Forsythia can reduce the growth rate of Enterobacter cloaves bacteria and increase the diameter of their inhibitory circle (Liu et al., 2020a). TCM has antibacterial potential such as low toxicity, low drug resistance and abundant resources. Using different TCM active ingredients with the same antibiotic has a synergistic effect on drug-resistant bacteria. The same Chinese herbal ingredient can also have a synergistic antibacterial effect with different antibiotics. This is the reason why we hope to solve the problem of bacterial resistance through TCM, and also provide an important theoretical basis for finding alternative methods for drug-resistant bacteria. The combination of TCM and antibiotics has become a new trend in antibacterial treatment. In-depth study of the synergistic bacteriostatic effect of active ingredients of TCM and antibiotics *in vivo* and their synergistic mechanism may become an important research direction in the future.

## 6 Conclusions and prospects

In conclusion, compared with the traditional antibiotic treatment mode, TCM has the following four advantages: it can reduce, delay or even reverse bacterial drug resistance, which has a sensitive effect on existing antibacterial drugs, and can greatly reduce the eradication rate of drug-resistant bacteria when used in combination with antibiotics; It can inhibit or kill drug-resistant bacteria, and has a good antibacterial effect on drug-resistant bacteria; It can improve immunity, improve the body’s disease resistance as a whole, fully balance the body’s own state-adverse reactions of chemical antibacterial drugs-pathogenic bacteria, and play an overall regulatory role; Easy access to materials and treatment costs are much lower than Western medicines, which can reduce the burden on patients. Due to the difficulty and long cycle of new antibacterial drugs, the use of TCM to treat drug-resistant bacteria is a new direction to solve the treatment of drug-resistant bacteria, but it must also be noted that the drug screened *in vitro* and *in vivo* are re-evaluated. Moreover, there are still some drug-resistant bacteria such as Pseudomonas aeruginosa and Acinetobacter baumannii for less TCM treatment, and the theoretical basis of TCM for the treatment of drug-resistant bacteria is not yet solid, so the efficacy of pan-drug-resistant bacteria pneumonia can be studied from TCM, theoretical discussion can be strengthened, the advantages of TCM can be given full play, and TCM and western medicine can be combined to improve the cure rate.

## Author contributions

PX, RS, and NL provided the writing of articles. SD and XK organized tables and figures. MT, Xiaoyi Zhao, and ZJ revised the article and put forward key suggestions. YC provided financial support. All authors contributed to the article and approved the submitted version.
